# Effect of Vitrification on Lipidomics in Porcine Cumulus–Oocyte Complexes After In Vitro Maturation

**DOI:** 10.3390/cells15080716

**Published:** 2026-04-18

**Authors:** Xinyu Huang, Zhen He, Decai Xiang, Jing Fu, Xuemei Li, Junyu Jiang, Guobo Quan, Guoquan Wu, Baoyu Jia

**Affiliations:** 1 Key Laboratory for Porcine Gene Editing and Xenotransplantation in Yunnan Province, College of Veterinary Medicine, Yunnan Agricultural University, Kunming 650201, China; 18161412507@163.com (X.H.); lxm19187386050@163.com (X.L.); 13987988411@163.com (J.J.); 2 National Regional Genebank (Yunnan) of Livestock and Poultry Genetic Resources, Yunnan Provincial Engineering Laboratory of Animal Genetic Resource Conservation and Germplasm Enhancement, Yunnan Animal Science and Veterinary Institute, Kunming 650224, China; 18463287165@163.com (Z.H.); askalm@163.com (D.X.); fj11260027@163.com (J.F.); waltq20020109@163.com (G.Q.)

**Keywords:** oocytes, cumulus cells, vitrification, lipidomics

## Abstract

**Highlights:**

**What are the main findings?**

- Vitrification disturbs lipid metabolism in porcine oocytes and CCs, which mainly affects glycerophospholipid metabolism, fat digestion and absorption and cholesterol metabolism.
- Lysophosphatidylcholine supplementation during IVM reduces oxidative stress and enhances the developmental potential of vitrified porcine GV oocytes.

**What are the implications of the main findings?**

- This study elucidates the metabolic mechanism underlying cryoinjury in oocytes and CCs, deepening the understanding of lipid metabolism disorders caused by vitrification.
- It provides a theoretical basis for optimizing oocyte vitrification through lipid regulation.

**Abstract:**

Due to its high efficiency and safety, oocyte vitrification finds broad application in many fields of life sciences, such as clinical assisted reproduction and conservation of animal genetic resources. However, vitrification may cause cellular damage and reduce the quality of oocytes and their cumulus cells (CCs), which could be closely related to disorders in lipid metabolism. At present, the impact of vitrification upon the lipid profile of oocytes and CCs has not been systematically elucidated. In this study, we used porcine germinal vesicle cumulus–oocyte complexes (COCs) as a model to analyze their lipid characteristics after vitrification and in vitro maturation (IVM), utilizing untargeted lipid metabolomics. Our results showed that an overall count of 37 down-regulated and 8 up-regulated differential lipids was identified in the vitrified oocytes. Pathway analysis confirmed the enrichment in glycerophospholipid metabolism and fat digestion and absorption, etc. Combined with transcriptomic analysis, three enriched pathways were revealed, including the AMPK signaling pathway, metabolic pathways, and fatty acid elongation. On the other hand, a total of four down-regulated and eight up-regulated differential lipids were detected in the vitrified CCs. Pathway enrichment implicated autophagy, glycerophospholipid metabolism, etc. A joint analysis of metabolomic and transcriptomic data revealed four enrichment pathways, including cholesterol metabolism, fat digestion and absorption, regulation of lipolysis in adipocytes, and metabolic pathways. Notably, the supplementation of lysophosphatidylcholine during IVM attenuated oxidative stress, enhanced mitochondrial activity, and enhanced the viability and embryonic development of cryopreserved porcine oocytes. The results indicate that vitrification alters lipids in oocytes and CCs, and the supplementation of lipids plays a role in improving the quality of vitrified oocytes.

## 1. Introduction

Currently, assisted reproductive technology (ART) is one of the key strategies for resolving human infertility. Oocyte cryopreservation provides a feasible solution for preserving fertility in cancer patients and also offers a new way to improve fertility in those with ovarian dysfunction [1]. In contrast to traditional slow-freezing, vitrification is gaining popularity for oocyte cryopreservation as it avoids ice crystal formation and alleviates oocyte cryodamage [2]. However, research has demonstrated that the oocyte, following vitrification, exhibits low fertilization and developmental potential [3,4]. Vitrification leads to the phase transition of lipids in oocytes, reducing cryopreservation efficiency [5]. Pigs closely resemble humans in terms of genetics, pathology, anatomy, and nutrient metabolism, positioning them as invaluable for research in human disease, xenotransplantation, and evaluation of pharmaceutical agents [6]. Moreover, pigs have served as an effective model for human-assisted reproduction. Porcine oocytes, being large single cells with high water and lipid content and a complex cytoplasmic structure, face major challenges in cryopreservation [7]. Recent studies have shown that porcine germinal vesicle (GV) stage oocytes subjected to vitrification show greater benefits than those vitrified at the metaphase II (MII) stage [8,9]. However, the blastocyst development of vitrified oocytes remains lower than that of fresh oocyte counterparts [10], suggesting the presence of subcellular damage induced by vitrification.

The follicle is the ovary’s basic unit, and its normal development is essential to the reproductive performance of females [11]. The growth and development of follicles are regulated by many endocrine, paracrine and autocrine factors [12]. Cumulus cells (CCs) are the primary somatic cells in mature follicles [13]. These CCs provide a natural protective barrier for oocytes during cryopreservation, reducing oocyte cryodamage. CCs are essential for regulating female gamete development and meiotic maturation [14]. However, vitrification interrupts this communication between oocytes and CCs and then affects oocyte development [15].

Lipids, as core components of cell membranes and energy storage, are abundant in oocytes [16]. Cold stress increases membrane lipid peroxidation and disrupts membrane permeability and cellular energy metabolism, while lipid phase transition at low temperatures raises the risk of freezing damage [17,18]. Transcriptomic analysis has revealed that oocyte vitrification affects lipid metabolism and energy supply by interfering with the transcript levels of genes involved in lipid metabolism, resulting in an imbalance of lipid homeostasis and energy supply disorders [19]. Proteomic analysis has shown that the transcriptional levels of proteins linked to lipid transport and metabolism are altered in vitrified mouse oocytes, indicating that vitrification disrupts the balance of lipid transport [20].

While transcriptomic and proteomic studies have partially elucidated the mechanisms underlying cryodamage in oocytes, lipidomics has garnered substantial interest. This is because it enables the revelation of dynamic changes in lipids and their metabolic pathways across oocyte maturation and embryogenesis via high-throughput analysis [21]. However, the application of lipidomics in the reproduction field remains incompletely explored, and research on lipid-related cryodamage in vitrified oocytes is still regarded as being in an exploratory phase. In our study, GV porcine cumulus–oocyte complexes (COCs) were used as a model, and the untargeted lipidomics served to examine lipid abundance differences in oocytes and CCs after vitrification and in vitro maturation (IVM). Based on these bioinformatic findings, we attempted to enhance the competence of vitrified oocytes by supplementing the lipids during IVM. The aim was to elucidate how vitrification impacts lipid metabolism in COCs, providing a theoretical support for optimizing oocyte vitrification protocol through lipid regulation.

## 2. Materials and Methods

Unless specified otherwise, all biochemicals and chemical supplies used in this work were obtained from Sigma-Aldrich (Shanghai, China). These included tissue culture medium-199 (TCM-199), Dulbecco’s PBS (DPBS), and knockout serum replacement (KSR). CM-H_2_DCFDA, DAF-FM Diacetate, ThiolTracker, and MitoTracker™ Red CMXRos were obtained from ThermoFisher Scientific (Shanghai, China).

### 2.1. Oocyte Collection and Grouping

Porcine ovaries, obtained from juvenile hybrid gilts from a local slaughterhouse, were immersed in saline supplemented with penicillin and streptomycin, kept at 35–37 °C, then transported to the lab within 2 h and washed twice. Following ovarian cleaning, antral follicles (3–8 mm) were aspirated using a 20 mL syringe fitted with an 18-gauge needle to collect follicular contents, and moved to 50 mL conical tubes to allow for the natural sedimentation of COCs. COCs were rinsed two times using Tyrode’s lactate-HEPES-polyvinyl alcohol medium (TLH-PVA), then observed under a stereomicroscope (Olympus, Tokyo, Japan), and those with uniform cytoplasm and at least three layers of compacted CCs were selected.

### 2.2. Oocyte Vitrification and Thawing

BM consisted of DPBS with 20% (*v*/*v*) KSR. Prior to vitrification, COCs were rinsed in BM and exposed to 5% (*v*/*v*) EG for 10 min at 25 °C for pre-equilibration. Batches consisting of 10–15 COCs were then placed into a vitrification mixture containing 0.6 M sucrose, 50 mg/mL polyvinylpyrrolidone, and 35% (*v*/*v*) EG. After 20–30 s of immersion at 25 °C, the COCs were loaded onto the tip of a Cryotop carrier (Kitazato Biopharma, Shizuoka, Japan) and plunged into liquid nitrogen (LN2). For thawing, Cryotops bearing COCs were moved from LN2 into 1.0 M sucrose at 42 °C for 1 min, followed by being sequentially moved into 0.5 M and 0.25 M sucrose with 2.5 min per step. Afterward, the COCs were rinsed in BM for 5 min and underwent IVM.

### 2.3. Oocytes IVM and Assessment of Oocyte Survival and Nuclear Maturation

The IVM solution contained TCM-199 fortified with 10 ng/mL EGF, 3.05 mM D-glucose, 0.91 mM sodium pyruvate, 0.57 mM cysteine, 10% (*v*/*v*) porcine follicular fluid, and 0.5 μg/mL FSH and LH. For maturation, COCs were rinsed three times in IVM medium, then distributed in groups of 50–70 into 24-well plates holding 500 μL of this medium and incubated at 39 °C under 5% CO_2_ and 100% humidity over 42–44 h. Following IVM, CCs were detached via gentle repetitive aspiration in TLH-PVA with 0.1% (*w*/*v*) hyaluronidase. Morphological observation under a stereomicroscope was performed to evaluate the viability and nuclear maturation of porcine oocytes. Oocytes with ruptured zona pellucida, disappeared oolemma or brownish-yellow heterogeneous cytoplasm were defined as non-viable. In contrast, oocytes exhibiting an intact zona pellucida, bright oolemma, as well as homogeneous granular cytoplasm were considered viable [22,23]. Oocyte nuclear maturation was confirmed via first polar body emission.

### 2.4. Lipid Extraction and Lipidomics Sequencing

The obtained MII oocytes and their CCs were rapidly frozen in LN2 and subsequently preserved at −80 °C. Each experimental group included six independent biological replicates. Approximately 800 oocytes were used per replicate, with a total of at least 4800 oocytes in each group. To extract the lipid components from the thawed samples, 120 μL of pre-cooled 50% methanol was added to each, then shaken vigorously for complete homogenization. Afterward, the samples were allowed to rest at ambient temperature for 10 min. Subsequently, the collected extracts were incubated overnight at −20 °C to precipitate any proteins present. The following day, the extracts underwent centrifugation at 4000× *g* for 20 min, after which the liquid phase holding extracted lipids was carefully transferred to a 96-well plate. The lipid extracts were then diluted with a solution of isopropanol, acetonitrile, and water in a 2:1:1 volume ratio. From each sample, an equal volume of 10 μL was taken and pooled to create quality control (QC) samples. All specimens were stored at −80 °C pending LC-MS analysis. A TripleTOF 5600 Plus high-resolution tandem mass spectrometer (Sciex, Warrington, UK) was used for LC-MS analysis, with sample detection performed in both positive and negative ion modes. Reverse-phase chromatographic separation was achieved using a UPLC system (Sciex, UK) equipped with an ACQUITY UPLC T3 column (100 mm × 2.1 mm, 1.8 µm, Waters, UK). The mobile phase was a binary system of 0.1% formic acid in water and 0.1% formic acid in acetonitrile, using gradient elution under defined conditions. MS parameters included curtain gas pressure, ion source gas pressure, and interface heater temperature, along with other settings. Mass spectra were acquired using Information-Dependent Acquisition spanning 60–1200 Da, with a 4 s dynamic exclusion window. Mass calibration was performed every 20 injections, and QC samples were analyzed every 10 runs to monitor LC-MS stability.

### 2.5. Lipidomics Data Analysis

Conversion of raw mass spectrometry data into the interpretable mzXML format was accomplished using ProteoWizard’s MSconvert (V3.0) software. XCMS was utilized for peak extraction and quality control procedures, and subsequently, CAMERA was applied to annotate adduct ions for the extracted features. The MetaX (V1.4.2) software was used for lipid identification by aligning primary mass spectra with public databases and cross-referencing secondary mass spectra using the internal reference standard database. The Kyoto Encyclopedia of Genes and Genomes (KEGG) and LIPID MAPS databases were employed to annotate candidate lipids and thereby illuminate their physicochemical attributes and biological functionalities. The identification of differential lipids was achieved through univariate analysis and multivariate analysis (Principal Component Analysis (PCA), Partial Least Squares Discriminant Analysis (PLS-DA) with variable importance in projection [VIP] scores). Significantly differential lipids were defined by the criteria: Fold Change (FC) > 1.2 or FC < 0.85, VIP >1, and *p* < 0.05. For lipid quantification, raw intensities of all lipids extracted by XCMS were imported into metaX. Missing values were filtered (excluding lipids absent from >50% of QC samples or >80% of experimental samples) and imputed using the K-nearest neighbors algorithm. The Probabilistic Quotient Normalization method approach was utilized for data normalization purposes, followed by QC-based robust spline correction. Unstable lipids (ions with >30% coefficient of variation in QC samples) were removed. Secondary mass spectra were further validated by matching against the in-house standard spectral library to confirm lipid identities. On the other hand, conjoint analysis was conducted using lipidomic data and formerly disclosed transcriptomic data, based on the co-enriched KEGG pathway.

### 2.6. PA and Embryo Culture In Vitro

MII oocytes were pre-incubated in activation solution for 30 s before rapid transfer to a 1 mm fusion chamber coated with activation solution, followed by exposure to 130 V/mm direct current pulse for 80 μs, using a Cell Fusion Machine (Budapest, Hungary). Following activation, oocytes were incubated for 4 h in porcine zygote medium-3 (PZM-3) supplemented with 10 μg/mL cycloheximide and 5 μg/mL cytochalasin B, followed by transfer to fresh PZM-3 and culture at 39 °C in 5% CO_2_ with full humidity. Cleavage was evaluated at 48 h, and blastocyst formation was determined at 144 h.

### 2.7. Measurement of Oxidative Stress and Mitochondrial Activity

For measurement of ROS levels, oocytes were washed three times with DPBS containing 0.1% (*w*/*v*) PVA (DPBS-PVA), and subsequently incubated for 20 min at 39 °C in DPBS-PVA containing 10 μM CM-H_2_DCFDA. After three rinses with DPBS-PVA, labeled oocytes were imaged via confocal laser-scanning microscope (Nikon A1, Nikon, Tokyo, Japan). Quantitation of fluorescent signals was conducted employing ImageJ (1.47v, National Instituted of Health, Bethesda, MD, USA) software. Similarly, nitric oxide (NO), glutathione (GSH) and mitochondrial activity were stained with 5 μM DAF-FM Diacetate, 10 μM ThiolTracker Violet and 200 nM MitoTracker™ Red CMXRos, respectively. The operation procedure was the same as above.

### 2.8. Statistical Analysis

Data regarding survival rate, maturation rate, cleavage rate, blastocyst rate, blastocyst cell number and relative fluorescence intensity were assessed using an independent-sample *t*-test with SPSS 27.0 software (SPSS Inc., Chicago, IL, USA). Data are presented as the estimated mean ± SEM, and *p* < 0.05 was considered significant. Univariate analysis of FC and Student’s *t*-test, combined with the variable importance in the projection (VIP) derived from the multivariate statistical PLS-DA model, was applied to screen differentially expressed lipids. Lipids satisfying the following criteria were defined as significantly altered differential lipids: FC > 1.2 or FC < 0.85, VIP > 1, and *p* < 0.05. The graphical abstract was created with https://BioGDP.com [24].

## 3. Results

### 3.1. Lipid Fraction Analysis

**Figure 1:**
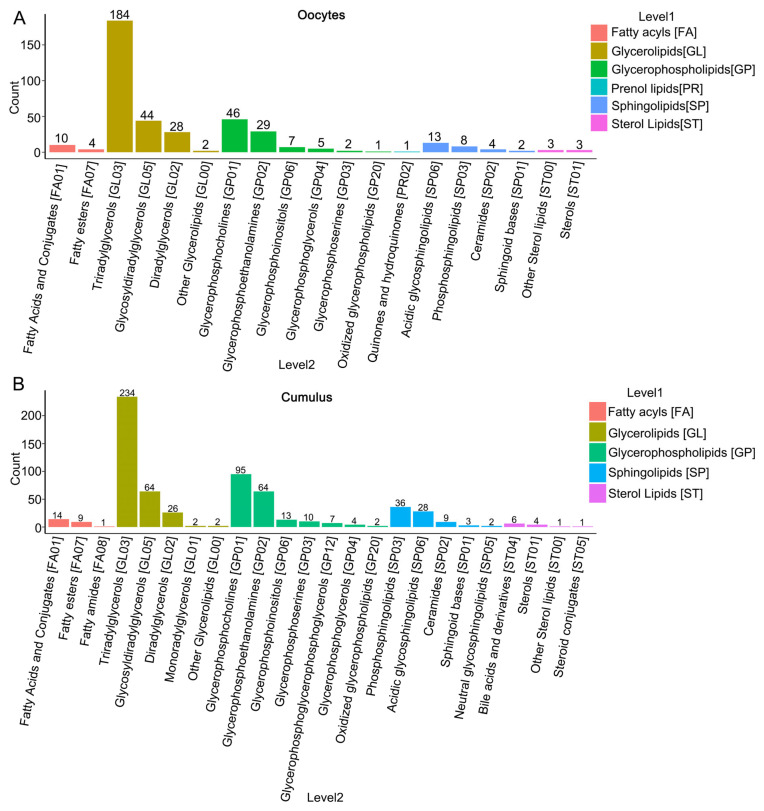
Analysis of lipid composition. (**A**) Different columns represent different lipid compositions in oocytes. (**B**) Different columns represent different lipid compositions in cumulus cells.

Lipidomics data acquired in both positive and negative ion patterns were integrated for bioinformatics analysis. In total, 396 lipids were identified in oocytes. Based on the LIPID MAPS classification system, lipids are categorized into eight categories, including sphingolipids (SP), prenol lipids (PR), glycerolipids (GL), glycerophospholipids (GP), sterol lipids (ST), fatty acyls (FA), saccharolipids (SL) and polyketides (PK) [25]. In this study, the metabolite composition of lipids in the oocytes was categorized into six groups, including 14 FA (3.54%), 258 GL (65.15%), 90 GP (22.73%), 1 PR (0.25%), 27 SP (6.82%) and 6 ST (1.52%) (Figure 1A, Supplemental Table S1). The main types of lipids in oocytes were GL and GP. These lipids could be divided into 30 lipid classes, with triacylglycerols (TG), phosphatidylcholines (PC), Diacylglycerol (DG), and lysophosphatidylcholine (LPC) being the major lipid classes in the oocytes (Figure S1, Supplemental Table S1).

Conversely, a total of 637 lipids were identified in CCs. These metabolites in CCs fell into five distinct categories, including 24 FA (3.77%), 328 GL (51.49%), 195 GP (30.61%), 78 SP (12.24%) and 12 ST (1.88%) (Figure 1B and Supplemental Table S2). The main types of lipids in CCs were also GL and GP. These lipids were categorized into 43 lipid classes, with TG, PC, and LPC as the primary classes in CCs (Figure S2 and Supplemental Table S2).

### 3.2. Lipid Differential Analysis of Vitrified and Fresh Porcine Germinal Vesicle Oocytes After In Vitro Maturation

**Figure 2:**
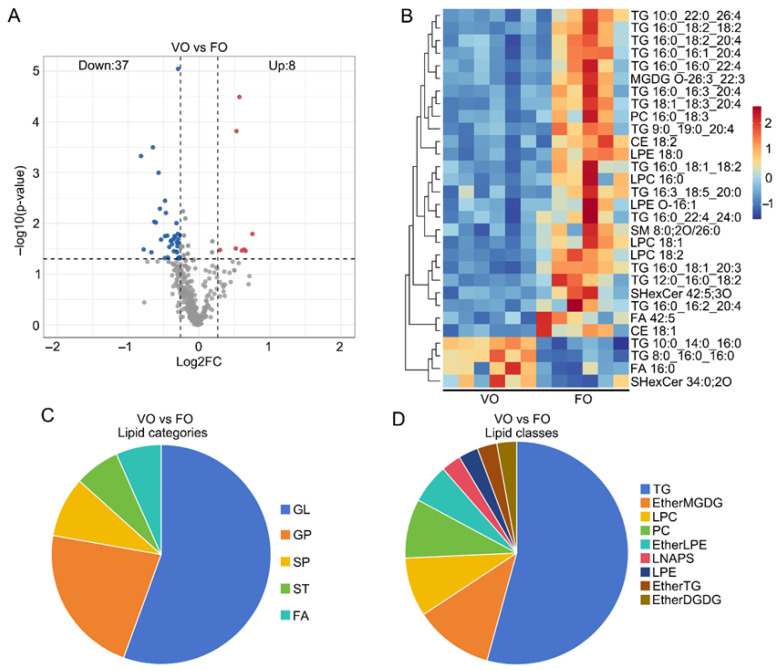
Analysis of differential lipid profile in vitrified and fresh porcine germinal vesicle (GV) oocytes after in vitro maturation (IVM). (VO: vitrified GV oocytes after IVM; FO: fresh GV oocytes after IVM.) (**A**) Volcano plots of biologically significant differential lipids were further screened based on VIP > 1 obtained using the PLS-DA model, and *p* < 0.05 and FC > 1.2 or FC < 0.85 obtained using univariate statistical analysis. Red, blue, and gray dots indicate up-regulated, down-regulated, and non-differential lipids, respectively. The dashed lines indicate the thresholds for |log_2_FC| ≥ 0.263 and −log_10_(*p*) ≥ 1.3 (*p* < 0.05). (**B**) Heat map of the top 30 differential lipids. (**C**) Pie chart of differential lipid categories in oocytes. (**D**) Pie chart of differential lipid classes in oocytes.

Differential lipids were analyzed in order to identify differential lipid markers in vitrified and fresh oocytes. Significant differential lipids were screened by PLS-DA analysis (Figure S3). Based on the screening criteria, there were a total of 45 differential lipids in vitrified oocytes compared to fresh ones, including 37 down-regulated and 8 up-regulated (Figure 2A, Supplemental Table S3). Clustered heatmap analysis showed marked variations in the expression patterns of candidate biomarkers across different experimental groups, while the expression patterns were similar in the samples within groups (Figure 2B, Supplemental Table S3). Further studies of differential lipids revealed that the main types of these differential lipids were GL and GP (Figure 2C). Among them, subclass TG in GL and subclasses PC and LPC in GP were enriched with more differential lipid ions (Figure 2D). The lipid ions LPC 18:2, PC 16:0_18:3, TG 12:0_16:0_18:2, TG 16:0_16:2_20:4, TG 18:1_18:3_20:4, LPC 18:1, and LPC 16:0 showed marked decreases in vitrified oocytes, whereas the lipid ions TG 10:0_14:0_16:0, TG 8:0_16:0_16:0 were significantly up-regulated.

### 3.3. Lipid Differential Analysis of Cumulus Cells Derived from Vitrified and Fresh Porcine Germinal Vesicle Oocytes After In Vitro Maturation

**Figure 3:**
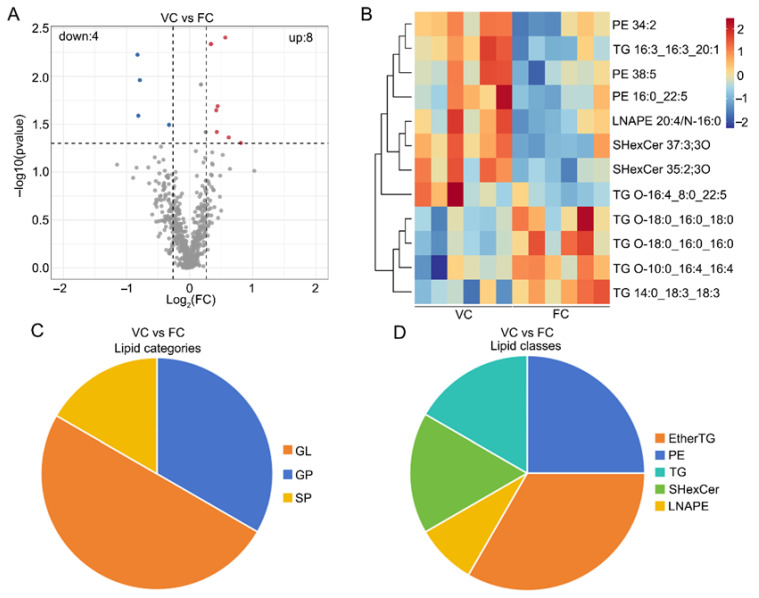
Analysis of differential lipid profile in cumulus cells (CCs) derived from vitrified and fresh GV oocytes after IVM. (FC: CCs derived from fresh immature oocytes after IVM; VC: CCs derived from vitrified immature oocytes after IVM.) (**A**) Volcano plots of biologically significant differential lipids were further screened based on VIP > 1 obtained using the PLS-DA model, and *p* < 0.05 and FC > 1.2 or FC < 0.85 obtained using univariate statistical analysis. Red, blue, and gray dots indicate up-regulated, down-regulated, and non-differential lipids, respectively. The dashed lines indicate the thresholds for |log_2_FC| ≥ 0.263 and −log_10_(P) ≥ 1.3 (*p* < 0.05). (**B**) Heat map of the top 30 differential lipids. (**C**) Pie chart of differential lipid categories in CCs. (**D**) Pie chart of differential lipid classes in CCS.

Similarly, lipids with significant differences were screened by PLS-DA analysis (Figure S4). A total of 12 lipids differed between vitrified and fresh CCs, with 4 showing down-regulation and 8 showing up-regulation. (Figure 3A, Supplemental Table S4). The expression patterns of potential biomarkers differed significantly between groups, but were similar within groups (Figure 3B, Supplemental Table S4). These differential lipids were primarily composed of GL and GP (Figure 3C). Among them, there was an enrichment of differential lipid ions in the subclass ether-linked triacylglycerol (EtherTG) and TG within GL, as well as phosphatidylethanolamine (PE) in GP. In vitrified CCs, the lipid ions TG O-18:0_16:0_16:0, TG O-18:0_16:0_18:0, TG 14:0_18:3_18:3, TG O-10:0_16:4_16:4 were significantly down-regulated, whereas the lipid ions PE 38:5, PE 34:2, TG 16:3_16:3_20:1, PE 16:0_22:5 and TG O-16:4_8:0_22:5 were significantly up-regulated.

### 3.4. KEGG Enrichment Analysis of Vitrified and Fresh Porcine Germinal Vesicle Oocytes After In Vitro Maturation

**Figure 4:**
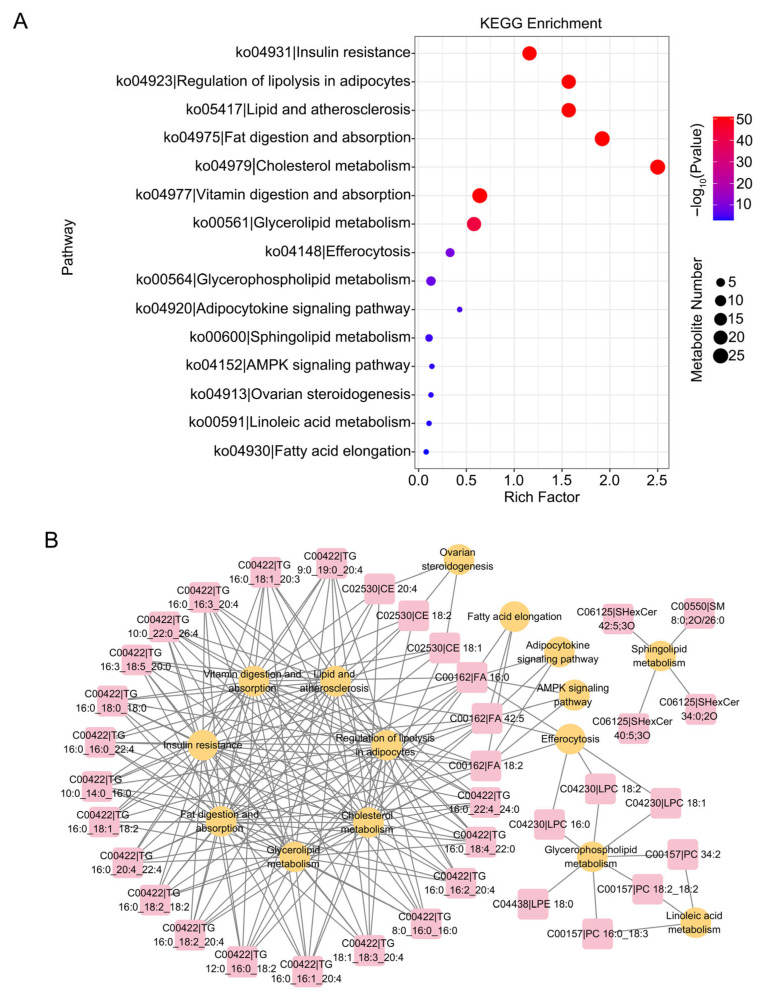
Differential lipid KEGG enrichment analysis of vitrified and fresh porcine GV oocytes after IVM. (**A**) Bubble plots of KEGG differential lipids. The enrichment factor represents the proportion of differential genes mapped to a given KEGG pathway relative to the overall count of genes annotated in that pathway. (**B**) Network diagrams of differential lipids and pathways of differential lipid enrichment. Yellow circles represent metabolic pathways, and red squares represent different lipids.

**Figure 5:**
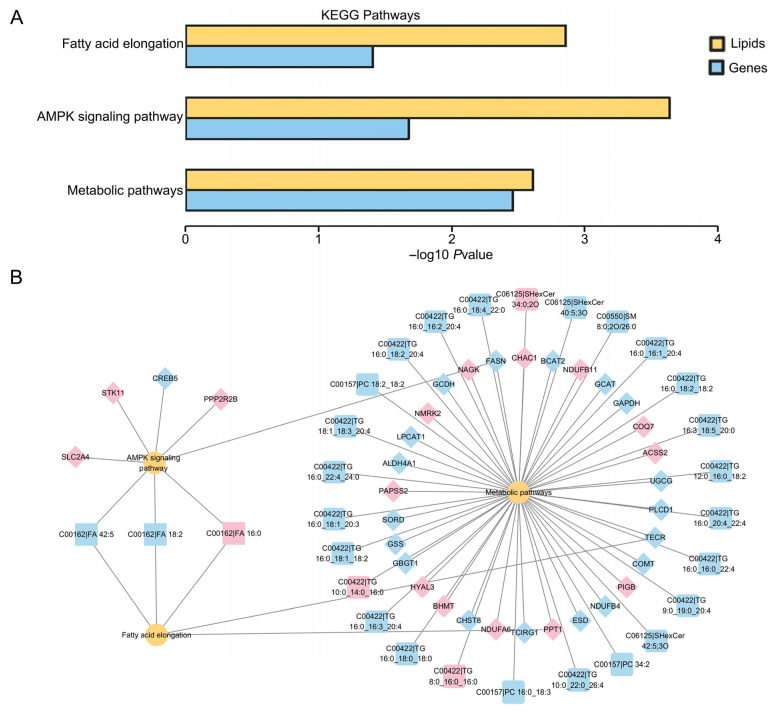
Combined transcriptome and lipidome analysis of vitrified and fresh porcine GV oocytes after IVM. (**A**) Pathways of differential gene and differential lipid co-enrichment in oocytes. (**B**) Changes in genes and lipids in signaling pathways. Yellow circles represent metabolic pathways, diamonds represent genes, squares represent lipids, red indicates elevated expression of genes or lipids, and blue denotes reduced levels.

To further investigate the function of differential lipids, we analyzed them for KEGG enrichment and identified 110 statistically significant pathways, predominantly related to cholesterol metabolism, glycerophospholipid metabolism, fat digestion and absorption, the AMPK signaling pathway, and glycerolipid metabolism (Figure 4A,B, Supplemental Table S5). Moreover, we performed an integrated analysis of transcriptome and lipidome data. The results revealed three co-enriched pathways, including fatty acid elongation, metabolic pathways, and the AMPK signaling pathway (Figure 5A,B).

### 3.5. KEGG Enrichment Analysis of Cumulus Cells Derived from Vitrified and Fresh Porcine Germinal Vesicle Oocytes After In Vitro Maturation

**Figure 6:**
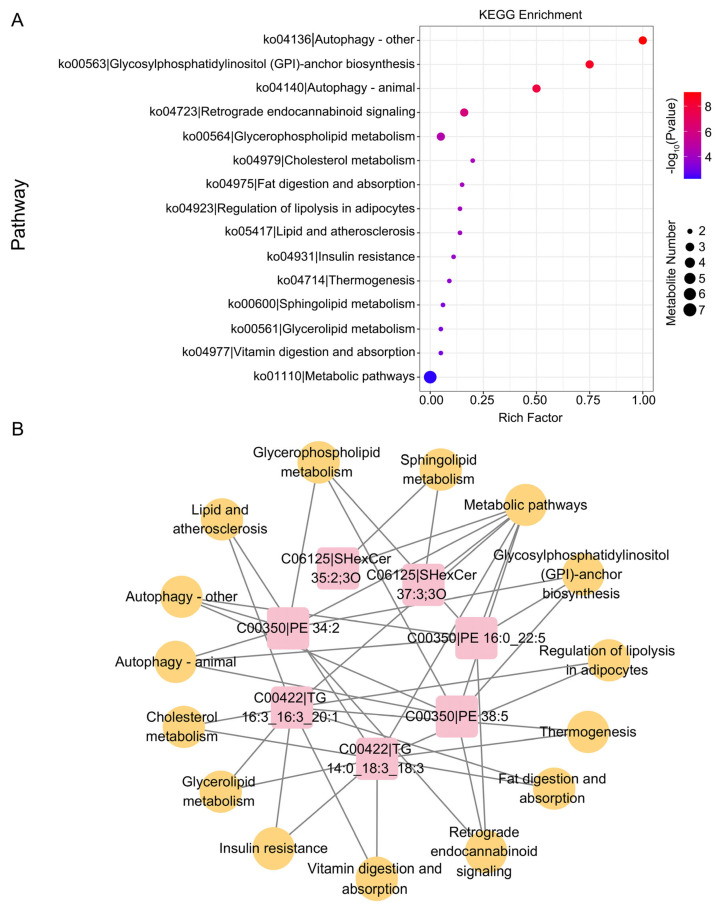
Differential lipid KEGG enrichment analysis of CCs derived from vitrified and fresh GV oocytes after IVM. (**A**) Bubble plots of KEGG differential lipids. The enrichment score is defined as the count of differential genes assigned to a given KEGG pathway divided by the total gene count in that pathway. A lower *p*-value corresponds to greater enrichment of that KEGG pathway. (**B**) Network diagrams of differential lipids and pathways of differential lipid enrichment. Yellow circles indicate metabolic pathways, and red squares indicate differential lipids.

**Figure 7:**
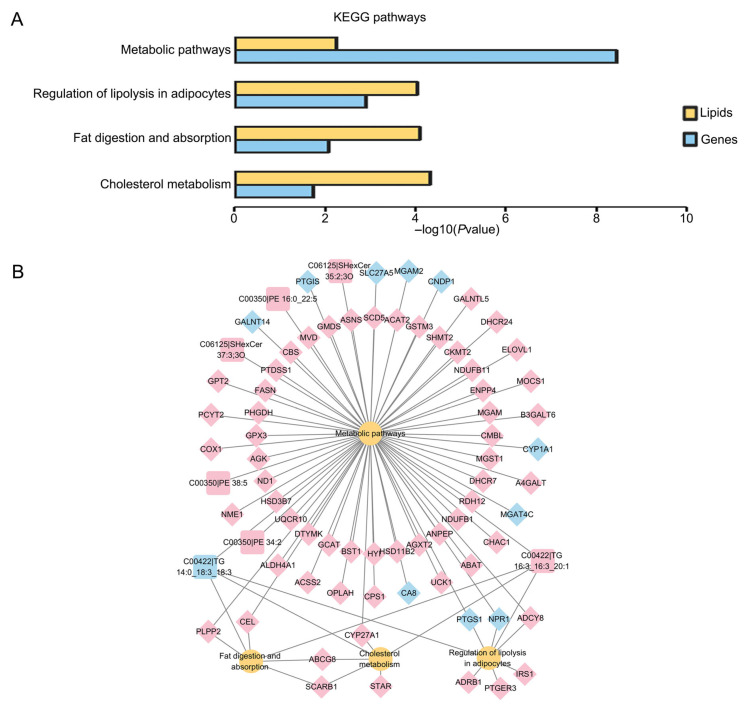
Combined transcriptome and lipidome analysis of CCs derived from vitrified and fresh GV oocytes after IVM. (**A**) Pathways of differential gene and differential lipid co-enrichment in cumulus cells. (**B**) Changes in genes and lipids in signaling pathways. Yellow circles represent metabolic pathways, diamonds indicate genes, squares represent lipids, red indicate up-regulated genes or lipids, and blue indicate down-regulated genes or lipids.

Based on the KEGG pathway enrichment, 18 significant differential pathways were obtained between vitrified and fresh CCs, such as metabolic pathways, autophagy, glycerolipid metabolism, glycerophospholipid metabolism and so on (Figure 6A,B, Supplemental Table S6). In addition, the integrative analyses of lipidomic and transcriptomic data revealed four co-enriched pathways, including cholesterol metabolism, metabolic pathways, regulation of lipolysis in adipocytes, and fat digestion and absorption (Figure 7A,B).

### 3.6. Lysophosphatidylcholine Improved the Quality of Vitrified Porcine Germinal Vesicle Oocytes After In Vitro Maturation

**Figure 8:**
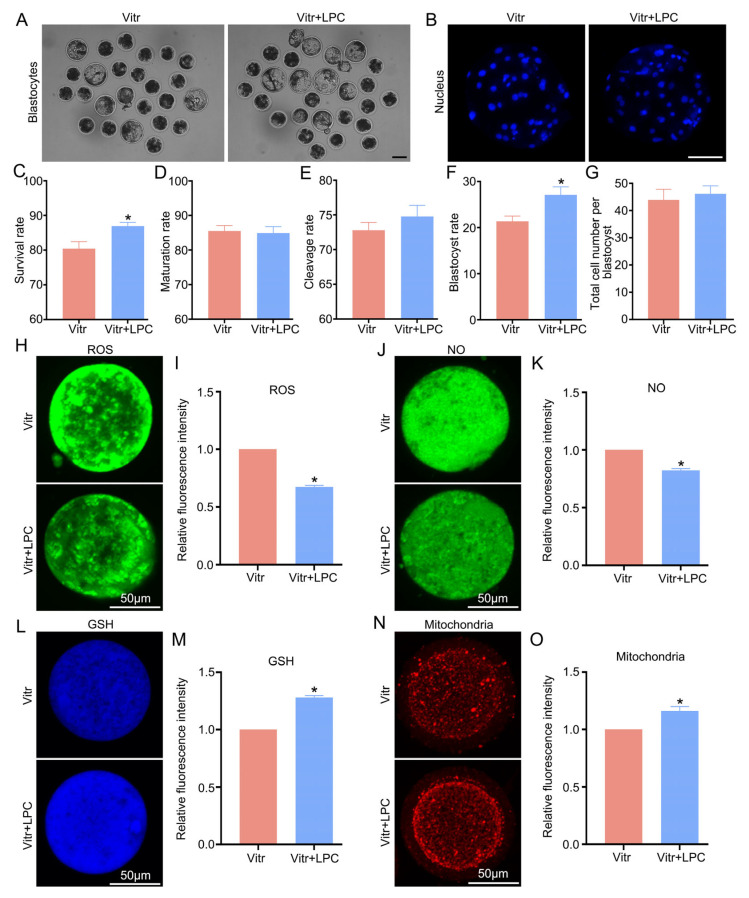
Lysophosphatidylcholine (LPC) improved the competence of vitrified porcine GV oocytes after IVM. (**A**,**B**) Hoechst 33342-stained nuclei in representative blastocysts obtained following parthenogenetic activation. (**C**–**G**): Oocyte survival (**C**), maturation rate (**D**), cleavage rate (**E**), blastocyst rate (**F**), and total cell number per blastocyst (**G**) in the Vitr and Vitr + LPC groups, respectively. (**H**–**O**): Representative images and relative fluorescence intensity of ROS, NO, GSH and mitochondrial fluorescence staining in the Vitr and Vitr + LPC, respectively. * Denotes a statistically meaningful intergroup difference (*p* < 0.05).

Prior work has shown that lysophosphatidylcholine (LPC) plays a critical regulatory role in oocyte maturation [26]. Our data indicate that LPC expression was down-regulated in VO compared to FO, which led us to conduct supplementation experiments to explore whether the addition of exogenous LPC during IVM could alleviate oocyte cryodamage induced by vitrification. In this experiment, the vitrified GV oocytes were matured within IVM medium containing 10 μM LPC. We found that LPC treatment significantly increased the post-warming viability of vitrified oocytes, but did not affect the maturation rate (Figure 8C,D). Moreover, the blastocyst formation rate was significantly higher in the vitrified oocytes that were treated with LPC, and there was a notable influence from LPC on cleavage rates and cell numbers of blastocysts (Figure 8A,B,E–G). In addition, the vitrified oocytes treated with LPC exhibited markedly lower amounts of ROS and NO, as well as significantly increased GSH content and mitochondrial activity (Figure 8H–O).

## 4. Discussion

Under physiological conditions, lipid metabolism serves as a critical energy source to support oocyte maturation and early embryo development [27]. Accumulating evidence has demonstrated that vitrification perturbs the lipid metabolic homeostasis in oocytes, consequently compromising their subsequent developmental potential. However, previous studies have not reported a comprehensive and detailed characterization of the dynamic lipid profiles in vitrified porcine COCs. In this study, we conducted untargeted lipid metabolomics to systematically identify the changes in lipid metabolism that occur in vitrified porcine GV oocytes and CCs following IVM. Our results are anticipated to offer conceptual understanding and empirical evidence to optimize the efficiency of oocyte vitrification protocols.

In this study, the reduced TGs observed in vitrified oocytes were overrepresented in multiple pathways closely associated with cellular energy metabolism, particularly the glyceride metabolism pathway. This pathway plays a central role in cellular energy storage, efficient energy supply, and maintenance of metabolic homeostasis. Furthermore, it serves as a key junction linking lipid and glucose metabolism, thereby exerting profound significance for the energy balance and material transformation [28]. In mammalian cells, free fatty acids (FFAs) can be converted into complex lipids, including TGs, phospholipids and sphingolipids, through metabolic processes such as elongation, desaturation, β-oxidation and peroxidation, thereby supporting intracellular lipid homeostasis [29]. Our study found that differentially expressed FFAs were also predominantly down-regulated and enriched in several key pathways, specifically the fatty acid elongation pathway, the AMPK signaling pathway, and the fatty acid degradation pathway. Based on the transcriptome data, the AMPK signaling pathway is also a significantly perturbed pathway in the blastocysts obtained from vitrified GV oocytes [19]. As a core energy regulatory system, the AMPK signaling pathway can be activated by physiological and pathological stressors in order to maintain cellular energy homeostasis by enhancing positive metabolic regulation [30]. Furthermore, an integrated lipidomics and transcriptomics analysis revealed that the differentially expressed fatty acid synthase (FASN) gene was enriched in the AMPK signaling pathway and the metabolism pathway. FASN, a key regulator of lipid synthesis and catabolism [31], was found to be down-regulated in the vitrified oocytes, which is consistent with the differentially expressed TG and FFA observed in this study. The transcriptional abundance of genes linked to lipid metabolism, such as SREBP1, FABP3, and PPARG, was found to be significantly altered in vitrified bovine oocytes [32]. A recent study showed that differentially expressed lipids, such as TG, DG, and PC, are enriched in pathways including AMPK signaling, fat digestion and absorption, and fatty acid metabolism in the vitrified bovine embryos through lipidomic analysis [33]. In a word, these differential lipids mainly participate in biological processes associated with cellular energy metabolism and oxidative stress, suggesting that vitrification of oocytes or embryos perturbed lipid metabolism and energy metabolism.

Cell membrane fluidity is correlated with membrane lipid composition [34]. GP is the main lipid component that constitutes the structure of eukaryotic cell membranes, with PC accounting for 40% to 50% of the total cellular phospholipid [35]. LPC generated by PC transformation is key to the metabolic intermediates of cell membrane phospholipids [26]. It can also promote the function of mature oocytes and the proliferation of CCs [36]. Previous studies have confirmed that vitrification impairs the fluidity and integrity of the plasma membrane in bovine oocytes. However, adding unsaturated fatty acids or cholesterol in vitro to regulate lipid levels can improve membrane fluidity and low-temperature tolerance of vitrified oocytes [37,38]. We found that the levels of GP, PC, and LPC decreased markedly within the vitrified oocytes. Furthermore, the differentially expressed PC were overrepresented in pathways linked to lipid metabolism, such as α-linolenic acid metabolism, arachidonic acid metabolism and glycerophospholipid metabolism. LC-MS analysis of lipidome changes in mouse oocytes has revealed that vitrification affects multiple lipid classes; however, mitigating changes in phospholipid and sphingolipid levels helps maintain oocyte membrane integrity [39]. In our previous study, several genes related to cell membrane function and metabolic homeostasis were found to be altered in the vitrified porcine GV oocytes after IVM [40]. This indicates that the compromised membrane integrity of vitrified oocytes is strongly tied to disrupted lipid metabolism.

Oocyte quality is tightly associated with the metabolic components of follicular fluid and the function of CCs [41]. For instance, CCs can supply energy for oocyte development through fatty acid β-oxidation [12]. Studies have shown that lipid metabolism disorders in CCs lead to impaired oocyte developmental function [42]. Currently, lipid metabolism in CCs is not completely understood. In our study, the differentially expressed TG in the vitrified CCs were overrepresented within metabolic pathways, such as cholesterol metabolism, lipid digestion and absorption, and regulation of lipolysis in adipocytes. The stearoyl-CoA desaturase (SCD) gene can regulate TG synthesis and FFA content [43]. We observed a significant up-regulation of the SCD5 gene in CCs through integrated transcriptomic analysis, suggesting that vitrification dramatically affects TG metabolism. On the other hand, PE is essential for cell membrane structure and also plays a part in cell division, membrane fusion, mitochondrial homeostasis and membrane protein biosynthesis [44,45,46]. We found that the up-regulated PE showed overrepresentation in pathways such as autophagy, glycerophospholipid metabolism, and cholesterol metabolism. CC autophagy selectively targets ATP citrate lyase to maintain citrate levels during oocyte maturation, thereby regulating normal follicular development [47]. This result suggests that autophagy levels in CCs could be elevated, induced by vitrification to maintain cellular homeostasis. In addition, alterations in the cholesterol metabolism pathway may affect oocyte survival, maturation outcomes, and subsequent fertilization capacity [48,49].

Lysophosphatidic acid (LPA), a lipid with growth factor-like activity, is highly enriched in follicular fluid, uterine secretions, serum, and plasma, and plays a key role in cellular development [50,51]. LPA is generated from LPC through conversion by autolysin [52]. As a secondary messenger, LPC is implicated in modulating oxidative stress, inflammation, and apoptosis, as well as being associated with follicular development [53,54]. LPA reportedly improves both survival and developmental potential of isolated follicles in the vitrified ovaries [55]. It is also found that supplementing with exogenous LPC could alleviate zearalenone-induced impairment and harm during the oocyte meiotic maturation [26]. So we next sought to confirm whether the LPC enhanced the competence of cryopreserved oocytes. Vitrification reduces the developmental capacity of oocytes, and the two main causes are elevated oxidative stress and abnormal mitochondrial function [56]. In our study, supplementation of LPC during IVM led to elevated levels in the percentages of vitrified oocytes that survived and formed blastocysts, indicating it might partially mitigate the negative impacts of vitrification. ROS exert opposing effects on cell fate decisions. Physiologically, ROS regulate the resumption and arrest of the meiotic cell cycle in mammalian oocytes [57]. However, vitrification of oocytes increases the production of intracellular ROS, resulting in oxidative injury [58]. Moreover, oxidative stress stimulates NO production, and its dysregulation results in lower oocyte quality and embryo implantation rates [59]. As an endogenous antioxidant, GSH maintains intracellular redox homeostasis and functions as a cytoplasmic maturation marker, predictive of oocyte fertilization capacity and subsequent developmental potential [60]. Our results suggested that LPC supplementation could mitigate oxidative stress and enhance antioxidant capacity in vitrified oocytes, indicated by reduced ROS and NO levels and increased GSH content. Lipid droplets in oocytes contribute to maintaining mitochondrial function. However, vitrification disrupts the interaction between lipid droplets and mitochondria, leading to mitochondrial dysfunction and lipid metabolism imbalance [61,62]. Our study has confirmed that LPC could potentially be used to improve mitochondrial function in vitrified oocytes.

## 5. Conclusions

In summary, the present study revealed changes in the lipidomics and corresponding regulatory pathways in porcine oocytes and CCs following vitrification and IVM. Several important lipids and key networks were found as well through integrating transcriptomic data. Moreover, adding LPC during IVM ameliorated oxidative stress and mitochondrial activity, and increased survival and embryonic development of vitrified oocytes. These findings will deepen our insight into the mechanisms driving oocyte cryoinjury and offer a conceptual foundation for refining oocyte vitrification protocols.

## Data Availability

The original contributions presented in this study are included in the article/Supplementary Materials. Further inquiries can be directed to the corresponding authors.

## Supplementary Materials

The following supporting information can be downloaded at: https://www.mdpi.com/article/10.3390/cells15080716/s1, Figure S1: Lipid classification of vitrified and fresh porcine germinal vesicle (GV) oocytes after in vitro maturation (IVM); Figure S2: Lipid classification of cumulus cells derived from vitrified and fresh GV oocytes after IVM; Figure S3: Differences in lipid profile of vitrified and fresh porcine GV oocytes after IVM; Figure S4: Differences in lipid profiles of cumulus cells derived from vitrified and fresh GV oocytes after IVM; Table S1: VOvsFO_Classification of lipid metabolites; Table S2: VCvsFC_Classification of lipid metabolites; Table S3: VOvsFO_Differential lipid metabolites; Table S4: VCvsFC_Differential lipid metabolites; Table S5: VOvsFO_KEGG_Enrichment; Table S6: VCvsFC_KEGG_Enrichment.

## Abbreviations

The following abbreviations are used in this manuscript:

IVM	In vitro maturation
ART	Assisted reproductive technology
CCs	Cumulus cells
COCs	Cumulus–oocyte complexes
FA	Fatty acyl
FFAs	Free fatty acids
GL	Glycerolipids
GP	Glycerophospholipids
GSH	Glutathione
GV	Germinal vesicle
KEGG	Kyoto Encyclopedia of Genes and Genomes
LPC	Lysophophatidylcholine
LPA	Lysophosphatidic acid
MII	Metaphase II
NO	Nitric oxide
PC	Phosphatidylcholines
PCA	Principal Component Analysis
PE	Phosphatidylethanolamine
PR	Prenol lipids
ROS	Reactive oxygen species
SL	Saccharolipids
SP	Sphingolipids
ST	Sterol lipids
TG	Triacylglycerols
