# Supplementary material for: Effect of Vitrification on Lipidomics in Porcine Cumulus–Oocyte Complexes After In Vitro Maturation
Source: Cells. 2026 Apr 18;15(8):716. doi: 10.3390/cells15080716 (PMC13114417; doi:10.3390/cells15080716)
Supplement: Supplementary file 1 [file cells-15-00716-s001.zip › Supplemental Figures.pdf]

Article

Effect of Vitrification on Lipidomics in Porcine Cumulus–Oocyte Complexes After In Vitro Maturation

Xinyu Huang [1,†], Zhen He [2,†], Decai Xiang [2], Jing Fu [2], Xuemei Li [1], Junyu Jiang [1], Guobo Quan [2], Guoquan Wu [2,*] and Baoyu Jia [1,*]

[1] Key Laboratory for Porcine Gene Editing and Xenotransplantation in Yunnan Province, College of Veterinary Medicine, Yunnan Agricultural University, Kunming 650201, China; 18161412507@163.com (X.H.); lxm19187386050@163.com (X.L.); 13987988411@163.com (J.J.)

[2] National Regional Genebank (Yunnan) of Livestock and Poultry Genetic Resources, Yunnan Provincial Engineering Laboratory of Animal Genetic Resource Conservation and Germplasm Enhancement, Yunnan Animal Science and Veterinary Institute, Kunming 650224, China; 18463287165@163.com (Z.H.); askalm@163.com (D.X.); fj11260027@163.com (J.F.); waltq20020109@163.com (G.Q.)

* Correspondence: wuguoquan1982@163.com (G.W.); jiabaoyu2009@163.com (B.J.)

† These authors contributed equally to this work.

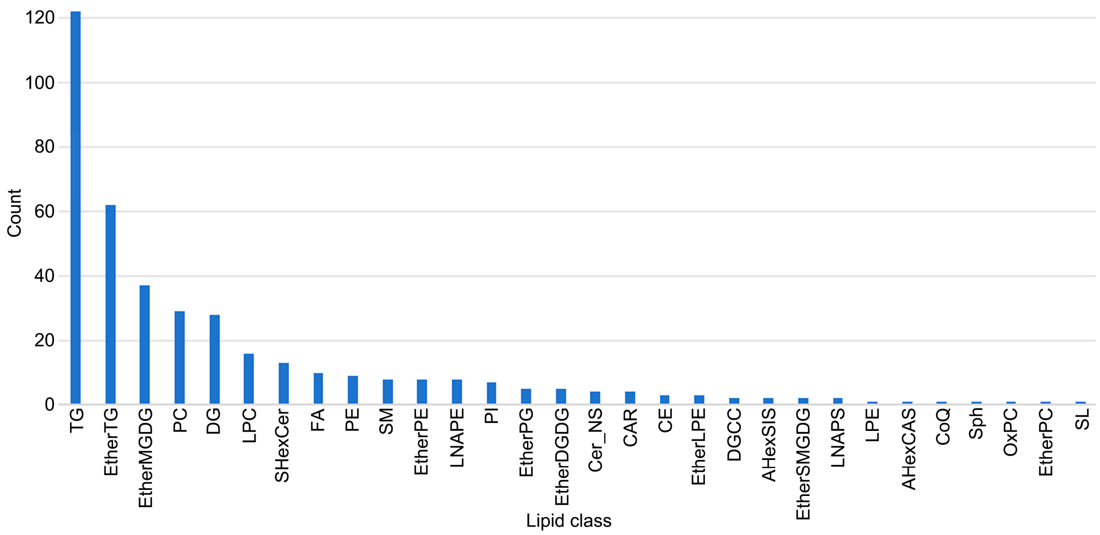

Figure S1. Lipid classification of vitrified and fresh porcine germinal vesicle (GV) oocytes after *in vitro* maturation (IVM).

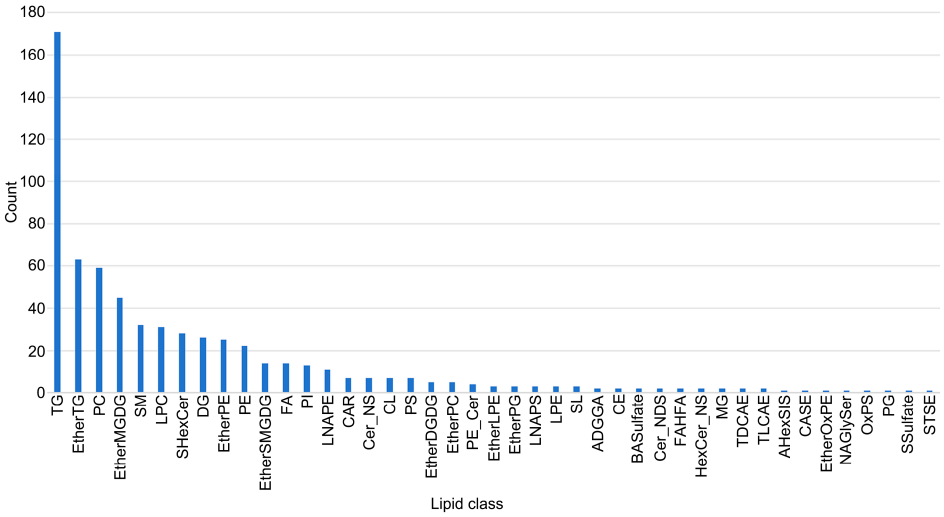

Figure S2. Lipid classification of cumulus cells derived from vitrified and fresh GV oocytes after IVM.

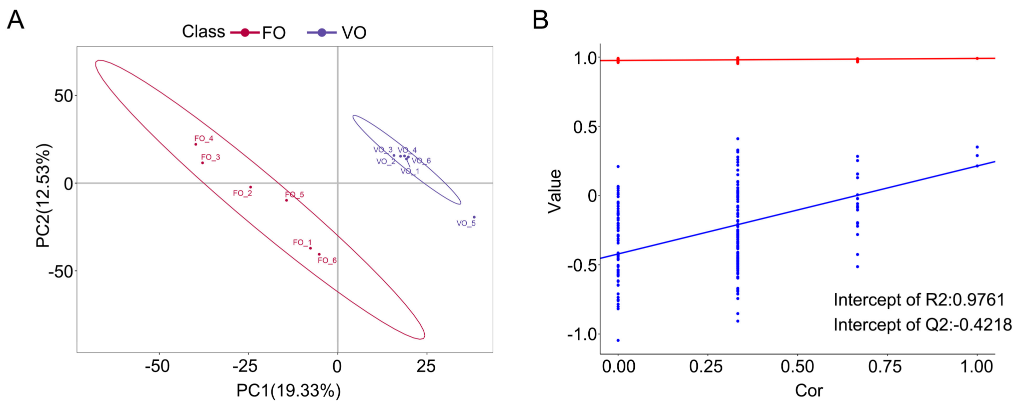

Figure S3. Differences in lipid profile of vitrified and fresh porcine GV oocytes after IVM. (A) Partial Least Squares Discriminant Analysis (PLS-DA). FO: Fresh GV oocytes after IVM. VO: Vitrified GV oocytes after IVM. (B) Permutation test of PLS-DA model.

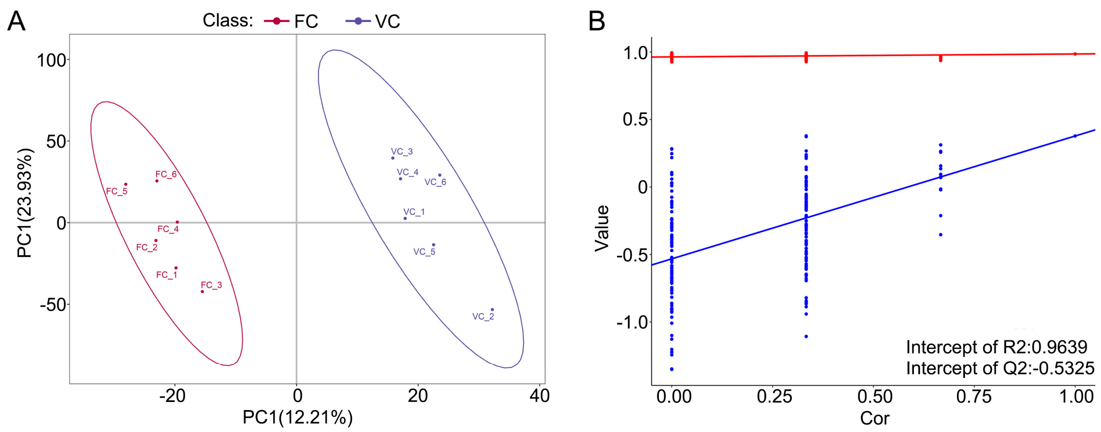

Figure S4. Differences in lipid profiles of cumulus cells derived from vitrified and fresh GV oocytes after IVM. (A) Partial Least Squares Discriminant Analysis (PLS-DA). FC: Fresh Cumulus cells after IVM of porcine immature oocytes. VC: Vitrified Cumulus cells after IVM of porcine immature oocytes. (B) Permutation test of PLS-DA model.

Supplemental Table S1 VOvsFO_Classification of lipid metabolites

Supplemental Table S2 VCvsFC_Classification of lipid metabolites

Supplemental Table S3 VOvsFO_Differential lipid metabolites

Supplemental Table S4 VCvsFC_Differential lipid metabolites

Supplemental Table S5 VOvsFO_KEGG_Enrichment

Supplemental Table S6 VCvsFC_KEGG_Enrichment
